# Paraoxonase-1 Is Not a Major Determinant of Stent Thrombosis in a Taiwanese Population

**DOI:** 10.1371/journal.pone.0039178

**Published:** 2012-06-18

**Authors:** Dong-Yi Chen, Chao-Yung Wang, Ming-Shien Wen, Tsong-Hai Lee, Yen Chu, Ming-Jer Hsieh, Shang-Hung Chang, Cheng-Hung Lee, Jian-Liang Wang, Chun-Chi Chen, Laing-Suei Lu, Ming-Ta Lee, San-Jou Yeh, Fun-Chiung Lin, I-Chang Hsieh

**Affiliations:** 1 Second Section of Cardiology, Department of Medicine, Chang Gung Memorial Hospital, Chang Gung University College of Medicine, Taoyuan, Taiwan; 2 Stroke Center and Department of Neurology, Chang Gung Memorial Hospital, Chang Gung University College of Medicine, Taoyuan, Taiwan; 3 Division of Thoracic and Cardiovascular Surgery, Chang Gung Memorial Hospital, Chang Gung University College of Medicine, Taoyuan, Taiwan; 4 Institute of Biomedical Sciences, Academia Sinica, Taiwan; University of Virginia Health System, United States of America

## Abstract

**Background:**

Clopidogrel is a prodrug that undergoes in vivo bioactivation to show its antiplatelet effects. Recent studies have shown that cytochrome P450 (CYP), ATP-binding cassette transporters (ABCB1), and paraoxonase-1 (PON1) play crucial roles in clopidogrel bioactivation. Here, we aim to determine the effects of genetic polymorphisms of *CYP* (*CYP* 2C19*2, *CYP* 2C19*3, and *CYP* 2C19*17), *ABCB1* (*ABCB1* 3435C>T, *ABCB1* 129T>C, and *ABCB1* 2677G>T/A), and *PON1* (*PON1* Q192R, *PON1* L55M, and *PON1* 108C>T) on the development of stent thrombosis (ST) in patients receiving clopidogrel after percutaneous coronary intervention (PCI).

**Methods and Results:**

We evaluated the incidence of ST (0.64%) in 4964 patients who were recruited in the CAPTAIN registry (Cardiovascular Atherosclerosis and Percutaneous TrAnsluminal INterventions). The presence of genetic polymorphisms was assessed in 20 subjects who developed ST after aspirin and clopidogrel therapy and in 40 age- and sex-matched control subjects who did not develop ST, which was documented after 9 months of angiographic follow-up. ST was acute in 5 subjects, subacute in 7, late in 7, and very late in 1. The presence of *CYP* 2C19*2 allele was significantly associated with ST (adjusted odds ratio [ORadj]: 4.20, 95% confidence interval [CI], 1.263–9.544; *P* = 0.031). However, genetic variations in *PON1* and *ABCB1* showed no significant association with ST.

**Conclusion:**

We conclude that in a Taiwanese population, *PON1* Q192R genotype is not associated with ST development after PCI. However, the presence of *CYP* 2C19*2 allele is a risk factor for ST development after PCI.

## Introduction

Dual antiplatelet therapy with aspirin and clopidogrel, an adenosine diphosphate (ADP)-P2Y12 receptor antagonist, has become the standard treatment for patients with coronary artery disease (CAD) who undergo percutaneous coronary intervention (PCI) with stenting [1], [2]. However, one of the shortcomings of clopidogrel therapy is high interindividual variability of its antiplatelet response [3], [4], [5]. Inadequate platelet inhibition may result in stent thrombosis (ST) and increase the frequency of cardiovascular events [6], [7], [8]. Clopidogrel is a prodrug that requires bioactivation, i.e., in vivo conversion into an active metabolite, to show its antiplatelet effects. Pharmacokinetic and pharmacodynamic studies have shown that the bioconversion of clopidogrel is a 2-step process and is mediated by different enzymes such as ATP-binding cassette transporters (ABCB1), hepatic cytochrome P450s (CYPs), and esterase paraoxonase-1 (PON1) [9].

Previous studies in young healthy volunteers receiving clopidogrel have shown that a loss-of-function mutation in *CYP*, which yields the *CYP* 2C19*2 allele, is associated with a marked decrease in platelet responsiveness to clopidogrel [10]. Other studies have shown that the presence of *CYP* 2C19*2 is significantly associated with a low rate of clopidogrel bioactivation [11], [12], [13]. Clinical studies conducted in a large group of patients with cardiovascular conditions who underwent PCIs have also confirmed that *CYP* 2C19*2 is associated with diminished clopidogrel responsiveness and increased frequency of major adverse cardiovascular events, such as recurrent myocardial infarction, ST, and long-term mortality [14], [15], [16], [17], [18].

However, the *CYP* 2C19 polymorphisms are only partly responsible for the low rates of clopidogrel bioactivation and its role in cardiovascular outcome is still controversial [19]. Previous studies showed that genetic variations in *CYP* 3A4-encoding CYP enzymes, which contribute to clopidogrel bioactivation [20], *ABCB1*, which modulates clopidogrel absorption [21], and *PON1*, may play major roles in clopidogrel metabolism.

PON1 is an arylesterase found in the liver and is involved in cell-mediated oxidation of high- and low-density lipoproteins (HDL and LDL) and inhibition of atherosclerotic processes. Previous studies revealed a controversial associated between PON1 polymorphism and coronary artery disease [22], [23]. A recent study showed that a common *PON1* polymorphism, Q192R, is associated with clopidogrel bioactivation [24]. Bouman et al. showed that PON1 is the rate-limiting enzyme in the second step of clopidogrel bioactivation, namely, hydrolytic cleavage of 2-oxo-clopidogrel to form the active thiol metabolite. They performed a case-cohort study of 112 individuals and showed that the *PON1* Q192R polymorphism, rather than *CYP* 2C19, is the major determinant of clopidogrel bioactivation.

Recent studies do not associate platelet responsiveness and the risk of ST development with *PON1* Q192R polymorphism [25], [26], [27], [28]. The reason for the different results obtained in various studies on the influence of PON1 in clopidogrel bioactivation is unclear. We hypothesize that this could be because of the differences in the ethnicity and genetic background of the study subjects. There might be some differences in *PON1* or *CYP* genotype distribution between Asian and Caucasian populations [29], [30]. Because different genotype prevalences can lead to different clinical effects, we investigated the effects of gene polymorphisms of *PON1* (*PON1* Q192R, *PON1* L55M, and *PON1* 108C>T), *ABCB1* (*ABCB1* 3435C>T, *ABCB1* 129T>C, and *ABCB1* 2677G>T/A), and *CYP* (*CYP* 2C19*2, *CYP* 2C19*3, and *CYP* 2C19*17) on the development of ST in Taiwanese patients receiving clopidogrel after PCI.

## Materials and Methods

### Ethics Statement

Written informed consent was obtained from 26 patients with ST and from 40 patients who served as controls. The study was approved by the Chang Gung Medical Foundation Institutional Review Board and conforms to the ethical guidelines of the Helsinki declaration.

### Study Population and Study Principle

Since November 1995, we have been registering CAD patients in the CAPTAIN registry (Cardiovascular Atherosclerosis and Percutaneous TrAnsluminal INterventions). To date, 4964 patients have been enrolled in the registry. We enroll only those patients who have undergone PCI with stenting and have been followed up regularly at the outpatient clinic. The overall follow-up rate is 72%. Long-term follow-up data up to June 2011 were obtained from the outpatient clinics.

For this study, we screened the data of ST patients from the CAPTAIN registry. These definition of ST complied with the consensus criteria definition by the Academic Research Consortium (ARC), and the cases of ST were further classified as acute (within 24 h after stent implantation), subacute (1–30 days), late (>30 days to 1 year) and very late (>1 year) [31]. We also screened and analyzed the data of age-, gender-, and risk factor-matched subjects without ST from the registry. PCI and post-PCI treatment procedures complied with current standard guidelines [1].

The patients in the normal control Han Chinese and Caucasian groups were randomly selected from the Cell and Genome Bank in Taiwan [32].

### Genotyping

Blood sampling was performed after PCI. DNA was extracted from 5 mL of blood using DNeasy blood kit (Qiagen) according to the manufacturer’s instructions. Sequencing of *PON1* Q129R (**rs662**), *CYP* 2C19*2 (**rs4244285**), *CYP* 2C19 *3 (**rs4986893**), *CYP* 2C19*17 (**rs12248560**), and *ABCB1* C3435T (**rs1045642**) was performed with a TaqMan assay by using an ABI Prism Sequence Detector 7000 (Applied Biosystems) according to the manufacturer’s protocols. Hardy–Weinberg equilibrium within each ethnic group was tested and was found to be nonsignificant for all gene polymorphisms (*P*>0.05). The genotyping results were reconfirmed by performing polymerase chain reaction (PCR) analysis and direct sequencing. The overall error rate was found to be less than 1%.

### Statistical Analysis

All variables are presented as mean ± standard deviation (SD) values and counts (in percentages). Categorical variables were compared using the χ^2^ test. The Kolmogorov–Smirnov test was used to check for normal distribution of continuous data. Continuous variables were evaluated using the Student’s *t*-test or one-way analysis of variance (ANOVA), as appropriate. Binary and polychotomous variables were examined using Fisher’s exact and χ^2^ tests. A multiple logistic regression model was used to test whether gene polymorphisms of *PON1* and *ABCB1* and *CYP* 2C19*2, *CYP* 2C19*3, and *CYP* 2C19*17 were independent predictors of ST. In addition to *PON1* Q192R and *CYP* 2C19*2, the other polymorphisms were also considered in the multivariable model and their details were entered as the number of risk alleles identified in the patients (0, 1, or 2) and by assuming a codominant model for the allele effect; all variables that differed (*P*<0.10) between ST and control subjects were also included in the multivariable model. A *P* value less than 0.05 was considered statistically significant. All statistical analyses were performed with Statistical Package for Social Sciences for Macintosh (SPSS for Mac; version 18; SPSS Institute).

## Results

### Subjects with Stent Thrombosis in the CAPTAIN Registry

In the CAPTAIN registry, we enrolled 4964 patients who underwent PCI with stenting from November 1995 to June 2011. The overall follow-up rate was 72%, and the incidence of ST was 0.64%. There were 32 cases of definite ST as defined by the ARC criteria among patients in the registry. Among these 32 patients, 6 refused to participate in the study, and 6 died before we could include them in the study. Thus, 20 ST subjects were enrolled as the ST group. For the control or non-ST group, we enrolled 40 age- and sex-matched subjects from the registry who had undergone PCI but did not develop ST, which was angiographically confirmed during a follow-up period of 9 months. All the 20 ST patients and 40 control patients received clopidogrel treatment after stent implantation for 9 months without discontinuation. Other P2Y12 antagonists were not used in the treatment. In 1 patient, ST occurred very late, i.e., 3 months after the physician recommended the discontinuation of clopidogrel after the 9-month treatment.

The baseline characteristics of patients of both the ST and non-ST groups are listed in Table 1. Clinical variables such as age, sex, hypertension and smoking history were well balanced between the 2 groups.

**Table 1:** Baseline clinical characteristics.

	*ST group	Non-ST group	*P* value
Variable	(n = 20)	(n = 40)	
**Age (mean), y**	60.3±8.9	59.0±7.5	0.564
**Male, n (%)**	17 (85%)	30 (75%)	0.384
**Hypertension, n (%)**	13 (65%)	18 (45%)	0.149
**Diabetes mellitus, n (%)**	9 (45%)	12 (30%)	0.132
**Smoking, n (%)**	11 (55%)	17 (42.5%)	0.369
**High sensitive** † **CRP** **(mg/L)**	14.2±18.9	18.9±37.1	0.597
**Total cholesterol (mg/dL)**	166.2±37.2	183.2±35.2	0.089
**Triglyceride (mg/dL)**	157.1±115.2	147.6±60.6	0.676
**Uric acid (mg/dL)**	5.7±1.3	6.2±1.7	0.243
**Ejection fraction (%)**	60.9±12.4	59.3±11.9	0.631

* ST, stent thrombosis.

† CRP, C-reactive protein.

Baseline clinical characteristics of the stent thrombosis and non-stent thrombosis groups.

Among the 20 ST patients, 5 (25%) received BMS and 15 (75%) received DES. Among the 40 control patients, 10 (25%) received BMS and 30 (75%) received DES. There was no angiographic coronary dissection in these patients.

### Correlation between the Risk of ST Development and Polymorphisms of *PON1*, *CYP*, and *ABCB1*

Among the 60 patients included in this study, 27 (45%) were *PON1* RR192 homozygous, 25 (41.7%) were QR192 heterozygous, and 8 (13.3%) were QQ192 homozygous carriers (Table 2). Among the 20 ST patients, 17 (85%) had at least 1 mutant allele of *PON1* Q192R, of which 10 were RR192 and 7 were QR192 carriers. Among the 40 non-ST subjects, 35 (87.5%) were carriers of at least 1 *PON1* mutant allele (17 were RR192 and 18 were QR192 carriers). There was no significant correlation between the *PON1* Q192R genotype and the risk of ST (Odds ratio [OR], 0.86, 95% confidence interval [CI], 0.372–2.821; *P* = 0.597). No significant associations were observed between ST development and the *PON1* L55M and *PON1* 108C>T polymorphisms (Tables 3).

**Table 2:** Genotype distributions in stent thrombosis (ST) and non-ST groups.

		*ST group	Non-ST group		
Genotypes		(n = 20)	(n = 40)	Total	*P* value
***CYP*** ** 2C19** * **2**	*2/*2 (AA)	4 (20%)	4 (10%)	8 (13.3%)	0.038
	wt/*2 (GA)	11 (55%)	12 (30%)	23 (38.3%)	
	wt/wt (GG)	5 (25%)	24 (60%)	29 (48.4%)	
***CYP*** ** 2C19** * **3**	AA	0	0	0	0.591
	AG	2 (10%)	6 (15%)	8 (13.3%)	
	GG	18 (90%)	34 (85%)	52 (86.7%)	
***CYP*** ** 2C19** * **17**	TT	0	0	0	-
	CT	0	0	0	
	CC	20 (100%)	40 (100%)	60 (100%)	
***PON1*** ** Q192R**	RR192	10 (50%)	17 (42.5%)	27 (45%)	0.760
	QR192	7 (35%)	18 (45%)	25 (41.7%)	
	QQ192	3 (15%)	5 (12.5%)	8 (13.3%)	
***PON1*** ** L55M**	AA	0	0	0	0.309
	AT	1 (5%)	2 (5%)	3 (5%)	
	TT	19 (95%)	38 (95%)	57 (95%)	
**PON1 108C>T**	TT	5 (25%)	8 (20%)	13 (21.7%)	0.873
	TC	10 (50%)	20 (50%)	30 (50%)	
	CC	5 (25%)	12 (30%)	17 (28.3%)	
***ABCB1*** ** C3435T**	TT	6 (30%)	5 (12.5%)	11 (18.3%)	0.188
	TC	9 (45%)	18 (45%)	27 (45%)	
	CC	5 (25%)	17 (42.5%)	22 (36.7%)	
***ABCB1*** ** T129C**	CC	0	0	0	0.509
	CT	1 (5%)	4 (10%)	5 (8.3%)	
	TT	19 (95%)	36 (90%)	55 (91.7%)	
***ABCB1*** ** G2677T**	TT	5 (25%)	6 (15%)	11 (18.3%)	0.544
	TA	3 (15%)	4 (10%)	7 (11.7%)	
	TG	6 (30%)	13 (32.5%)	19 (31.7%)	
	GA	2 (10%)	10 (25%)	12 (20%)	
	AA	0	2 (5%)	2 (3.3%)	
	GG	4 (20%)	5 (12.5%)	9 (15%)	

* ST, stent thrombosis.

The genotype frequencies of *PON1*, *CYP*, and *ABCB1* polymorphisms in the stent thrombosis (ST) and non-ST groups. A significant difference (*P* = 0.038) in genotype distribution between the ST and non-ST groups is seen only for *CYP* 2C19*2 and not for *PON1*, *CYP* 2C19*3, *CYP* 2C19*17, and *ABCB1* polymorphisms.

**Table 3:** Results of multivariable logistic regression for genotype carriers in predicting stent thrombosis.

	Carrier Odds Ratio*	*P* value
Variable	(95% †CI)	
***PON1*** ** Q129R rs662**	0.86 (0.372–2.821)	0.597
***PON1*** ** L55M rs854560**	1 (0.167–5.985)	0.423
***PON1*** ** 108 rs705379**	0.78 (0.23–2.627)	0.686
***CYP*** ** 2C19** * **2 rs4244285**	4.20 (1.263–9.544)	0.031
***CYP*** ** 2C19** * **3 rs4986893**	0.83 (0.315–4.451)	0.577
***CYP*** ** 2C19** * **17 rs12248560**	–	–
***ABCB1*** ** C3435T rs1045642**	2.32 (0.853–7.183)	0.112
***ABCB1*** ** 129 rs3213619**	1.541 (0.15–15.830)	0.341
***ABCB1*** ** 2677 rs2032582**	0.529 (0.14–2.008)	0.763

* Unadjusted odds ratios (ORs): *CYP* 2C19*2, OR, 4.50 (1.363–14.844), *P* = 0.028; *PON1* Q129R, OR, 0.74 (0.252–2.171), *P* = 0.697; *ABCB1* C3435T rs1045642, OR, 2.22 (0.674–7.293), *P* = 0.212.

† CI, confidence interval.

Regarding the *CYP* 2C19*2 genotype, 8 of the 60 patients (13.3%) were *2/*2 homozygous, 23 (38.3%) were wt/*2 heterozygous, and 29 (48.4%) were wt/wt homozygous carriers. Among the 20 ST patients, 15 (75%) were carriers of at least 1 *CYP* 2C19*2 mutant allele (4 *2/*2 carriers and 11 wt/*2 carriers). However, only 16 (40%) of the 40 non-ST patients were carriers of at least 1 *CYP* 2C19*2 mutant allele (4 *2/*2 carriers and 12 wt/*2 carriers). There was a significant correlation between the presence of *CYP* 2C19*2 variants and risk of ST development (OR, 4.2 [95% CI, 1.263–9.544]; *P* = 0.031).

Of the 20 ST patients, 5 had acute ST, 7 had subacute ST, 7 had late ST, and 1 had very late ST. The *PON1* Q192R mutant allele carrier rate was 80% (2 RR192 and 2 QR192 carriers) in 5 acute ST patients, 100% (4 RR192 and 3 QR192 carriers) in 7 subacute ST patients, 71.4% (3 RR192 and 2 QR192 carriers) in 7 late ST patients, and 100% (1 RR192 carrier) in 1 very late ST patient (Table 4). The *CYP2C19* mutant allele carrier rate was 80% (2 *2/*2 and 2 wt/*2 carriers) in 5 acute ST patients, 57.1% (4 wt/*2 carriers) in 7 subacute ST patients, 85.7% (2 *2/*2 and 4 wt/*2 carriers) in 7 late ST patients, and 100% (1 wt/*2 carrier) in 1 very late ST patient. There was no difference in genotype distribution of *PON1* Q192R and CYP2C19*2 between acute, subacute, late, and very late ST, with *P* values of 0.549 and 0.747, respectively.

**Table 4:** Genotype frequencies of acute, subacute, late, and very late stent thrombosis.

		Acute	Subacute	Late	Very late	*P* value
Genotypes		(n = 5)	(n = 7)	(n = 7)	(n = 1)	
***CYP*** 2C19*2	*2/*2 (AA)	2 (40%)	0	2 (28.6%)	0	0.549
	wt/*2 (GA)	2 (40%)	4 (57.1%)	4 (57.1%)	1(100%)	
	wt/wt (GG)	1 (20%)	3 (42.9%)	1 (14.3%)	0	
***PON1*** ** Q192R**	RR192	2 (40%)	4 (57.1%)	3 (42.8%)	1 (100%)	0.747
	QR192	2 (40%)	3 (42.9%)	2 (28.6%)	0	
	QQ192	1 (20%)	0	2 (28.6%)	0	

There was no difference in the genotype distribution of *PON1* Q192R and *CYP2C19* between acute, subacute, late, and very late ST, with a *P* value of 0.549 and 0.747, respectively.

**Figure 1:**
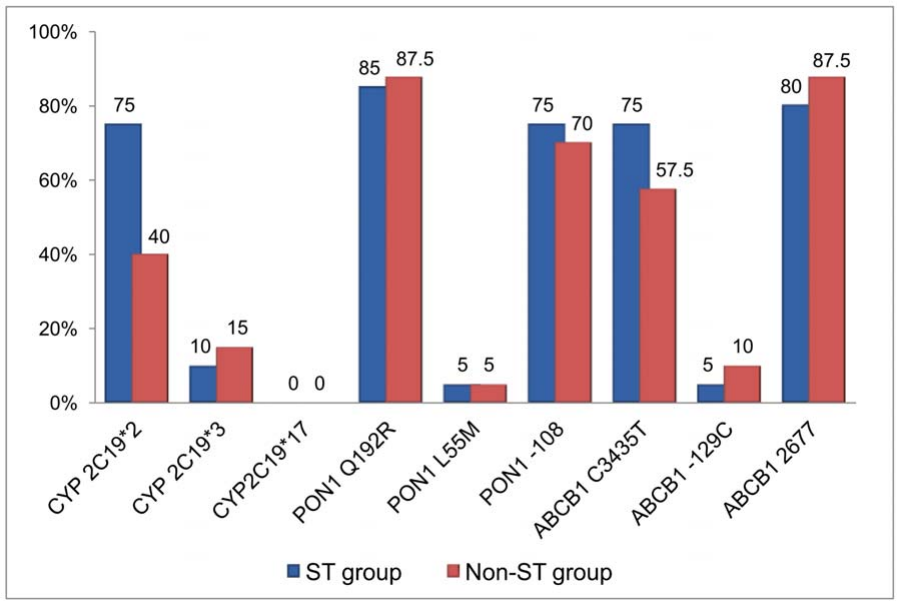
Percentage of mutant allele carriers of *CYP* 2C19 and *PON1* and *ABCB1* polymorphisms. Graph showing the percentage of mutant allele carriers for cytochrome P450 (*CYP*) and paraoxonase-1 (*PON1*) and ATP-binding cassette transporter (*ABCB1*) polymorphisms in stent thrombosis (ST) group (blue) and non-ST group (red). The carrier percentage for the *CYP* 2C19*2 mutant allele is significantly different between the ST and non-ST groups (75% vs. 40%; carrier odds ratio [OR 4.2; 95% confidence interval [CI], 1.263–9.544; *P* = 0.031), with a high carrier percentage in the ST group.

There were no significant correlations between the risk of ST development and the presence of *CYP* 2C19*3, *CYP* 2C19*17, *PON1* L55M, *PON1* 108C>T, *ABCB1* C3435T, *ABCB1* 129T>C, and *ABCB1* 2677G>T (Fig. 1). The mutant allele carrier rate for *CYP* 2C19*3 was 10% in the ST group and 15% in the non-ST group, with an OR of 0.83 (95% CI, 0.315–4.451; *P* = 0.577) for stent thrombosis. The carrier rate for *ABCB1* C3435T was 75% in the ST group and 57.5% in the non-ST group, with an OR of 2.32 (95% CI, 0.853–7.183; *P* = 0.112) for ST. The *CYP* 2C19*17, *PON1* L55M, *ABCB1* 129T>C mutant carrier rates in the study population were less than 5%; therefore, a larger study population is required to detect their effects on clopidogrel bioactivation.

## Discussion

In this study, we analyzed the effects of gene polymorphisms of *CYP* (*CYP* 2C19*2, *CYP* 2C19*3, and *CYP* 2C19*17), *ABCB1* (*ABCB1* 3435C>T, *ABCB1* 129T>C, and *ABCB1* 2677G>T/A), and *PON1* (*PON1* Q192R, *PON1* L55M, and *PON1* 108C>T) on the development of ST in patients receiving clopidogrel after PCI (Fig. 1). Our study shows that the *PON1* Q192R genotype is not associated with ST development after PCI in Taiwanese population. However, carriers of the *CYP* 2C19*2 allele showed an increased risk of ST development after PCI.

Clinical studies conducted in a large group of patients, mainly Caucasians, have confirmed that *CYP* 2C19*2 is associated with diminished clopidogrel responsiveness and increased frequency of key adverse cardiovascular events, such as recurrent myocardial infarction, ST, and long-term mortality [14], [15], [16], [17], [18]. The same result was reported by Luo et al. in a Chinese population [33]. Hence, our results were similar to those of previously reported studies.

Several recently published studies have failed to find the relationship between *PON1* Q192R polymorphism and antiplatelet responsiveness of clopidogrel [25], [26], [27], [28], [34]. Moreover, the relationships between *PON1* Q192R polymorphism and ST are still controversial [24], [26]. Our study showed that the *PON1* Q192R genotype is not associated with ST development after PCI in a Taiwanese population. This finding is in concordance with the results of a recent study by Sibbing et al. [26]; they studied a large cohort of 1524 patients and concluded that the *PON1* Q192R genotype is not associated with the risk of ST after coronary stenting by comparing 127 ST patients with 1439 controls in the same registry.

However, these results are different from the results of a recent study by Bouman et al. [24]. With in vitro metabolomics-profiling techniques, they identified PON1 as a key enzyme in the transformation of 2-oxo-clopidogrel to the active thiol metabolite. In this study, they compared *PON1* Q192R genotype frequencies in 41 ST patients and 71 controls and found significant association between *PON1* Q192R and ST.

The precise reasons for this discrepancy are still unclear. Most of the patients in the study by Bouman et al. and Sibbing et al. were Caucasians. Some researchers suggest that the differences in populations or study design may account for these differences in outcome [34]. Our study showed a different *PON1* Q192R genotype distribution in the control group (42.5% for RR192, 45% for QR192, and 12.5% for QQ192) as compared with the control groups reported by Sibbing et al. (8% for RR192, 39% for QR192, and 53% for QQ192) and Bouman et al. (18% for RR192, 47% for QR192, and 35% for QQ192). We have observed that the Taiwanese patients exhibit a high carrier rate for the *PON1* Q192R mutant allele.

Our study among the Han Chinese population showed no association between *PON1* Q192R and ST development. Further, both *PON1* L55M and *PON1* 108C>T polymorphisms showed no association with ST development in our study. Therefore, our study results support the fact that *PON1* polymorphisms do not contribute to ST development in populations with a different ethnic or genetic background.

Although we did not detect a significant association between *PON1* gene polymorphisms and ST development, we found differences in the genotype distribution of *PON1* and *CYP* 2C19*2 between Asian and Caucasian populations (Table 5). We performed the genotyping of 92 Han Chinese and 92 Caucasian people without CAD. The Han Chinese population showed an 80.1% carrier rate for the *PON1* Q192R mutant allele (44.5% for RR192 and 35.9% for QR192) whereas the Caucasian population showed a 35.9% carrier rate for the same polymorphism (4.4% for RR192 and 31.5% for QR192) (Table 4). The Han Chinese population also showed higher *CYP* 2C19*2 mutant allele carrier rate (12% for *2/*2 and 39.1% for wt/*2) than that of the Caucasian population (2.2% for *2/*2 and 22.8% for wt/*2). These findings showing significantly high carrier rates for *PON1* Q129R and *CYP* 2C19*2 in the Han Chinese population may have an important clinical impact [29], [30], [35], [36], [37]. The high *PON1* Q129R carrier rate in the Han Chinese population did not contribute to the rate of ST development. In contrast, the high carrier rate of *CYP* 2C19*2 in the Han Chinese population might have influenced the clopidogrel responses and cardiovascular outcome and warrant future investigation.

**Table 5:** Genotype frequencies in Asian and Caucasian populations.

		Normal Han Chinese population	Normal Caucasian population
Genotypes		(n = 92)	(n = 92)
***CYP*** 2C19*2	*2/*2 (AA)	11 (12.0%)	2 (2.2%)
	wt/*2 (GA)	36 (39.1%)	21 (22.8%)
	wt/wt (GG)	45 (48.9%)	69 (75.0%)
***CYP*** 2C19*3	AA	0	0
	AG	9 (10%)	0 (0%)
	GG	83 (90%)	92 (100%)
***PON1*** ** Q192R**	RR192 (GG)	41 (44.5%)	4 (4.4%)
	QR192 (AG)	33 (35.9%)	29 (31.5%)
	QQ192 (AA)	18 (19.6%)	59 (64.1%)
***ABCB1*** ** C3435T**	AA	13 (14.1%)	30 (32.6%)
	AG	45 (48.9%)	42 (45.7%)
	GG	34 (37.0%)	20 (31.7%)

The genotype frequencies of *CYP*, *PON1* Q192R, and *ABCB1* C3435T in normal Han Chinese population (*n* = 92) and normal Caucasian population (*n* = 92) are shown. Normal Han Chinese population has a higher *CYP* 2C19*2 mutant allele carrier rate (12% for *2/*2 and 19% for wt/*2) than that of the Caucasian population (2.2% for *2/*2 and 22.8% for wt/*2). The Han Chinese population also showed higher *PON1* Q192R mutant allele carrier rate (80.1%; 44.5% for RR192 and 35.9% for QR192) than that in Caucasian population (35.9%; 4.4% for RR192 and 31.5% for QR192).

### Limitations

Our study has some limitations. We had a limited sample size (n = 20 cases of ST) combined with the limited strength of the case-control design. Therefore, the identified ST cases in our study may not represent all ST cases, and we may have introduced a possible bias when selecting the control group. We did not use intravascular ultrasound (IVUS) during stent implantation. IVUS is a useful tool for detecting reduction in stent size and coronary dissection, which are powerful predictors of ST.

### Conclusion

In conclusion, we found that the *PON1* Q192R polymorphism was not associated with the risk of ST development after PCI in a Taiwanese population. However, the *CYP* 2C19*2 polymorphism remained a key risk factor for ST development in patients who had undergone PCI.
